# Cyclin-Dependent Kinases and CTD Phosphatases in Cell Cycle Transcriptional Control: Conservation across Eukaryotic Kingdoms and Uniqueness to Plants

**DOI:** 10.3390/cells11020279

**Published:** 2022-01-14

**Authors:** Zhi-Liang Zheng

**Affiliations:** 1 Department of Biological Sciences, Lehman College, City University of New York, Bronx, NY 10468, USA; zhiliang.zheng@lehman.cuny.edu; 2 Biology PhD Program, Graduate Center, City University of New York, New York, NY 10016, USA

**Keywords:** CDK, CTD phosphatase, RNA polymerase II, CTD code, transcription, cell cycle

## Abstract

Cell cycle control is vital for cell proliferation in all eukaryotic organisms. The entire cell cycle can be conceptually separated into four distinct phases, Gap 1 (G1), DNA synthesis (S), G2, and mitosis (M), which progress sequentially. The precise control of transcription, in particular, at the G1 to S and G2 to M transitions, is crucial for the synthesis of many phase-specific proteins, to ensure orderly progression throughout the cell cycle. This mini-review highlights highly conserved transcriptional regulators that are shared in budding yeast (*Saccharomyces cerevisiae*), *Arabidopsis thaliana* model plant, and humans, which have been separated for more than a billion years of evolution. These include structurally and/or functionally conserved regulators cyclin-dependent kinases (CDKs), RNA polymerase II C-terminal domain (CTD) phosphatases, and the classical versus shortcut models of Pol II transcriptional control. A few of CDKs and CTD phosphatases counteract to control the Pol II CTD Ser phosphorylation codes and are considered critical regulators of Pol II transcriptional process from initiation to elongation and termination. The functions of plant-unique CDKs and CTD phosphatases in relation to cell division are also briefly summarized. Future studies towards testing a cooperative transcriptional mechanism, which is proposed here and involves sequence-specific transcription factors and the shortcut model of Pol II CTD code modulation, across the three eukaryotic kingdoms will reveal how individual organisms achieve the most productive, large-scale transcription of phase-specific genes required for orderly progression throughout the entire cell cycle.

## 1. Introduction

The control of cell cycle is a vital process for cell proliferation in all eukaryotic organisms. The cell cycle from unicellular organisms, such as budding yeast (*Saccharomyces cerevisiae*), to multicellular organisms including plants and humans has shared some common features. These include four phases that progress from Gap 1 (G1) to DNA synthesis (S) and then to G2 and mitosis (M), completing a cell cycle. In the cell cycle, the G1 to S and G2 to M transitions are considered two key control points, with the former making an irreversible decision to enter the cycle, and the latter ensuring completion of faithful DNA synthesis before distributing genomes into new daughter cells [1]. In addition, the cyclin and cyclin-dependent kinases (CDKs) act as a core of the regulatory network that governs the progression of each phase in order [2,3]. Moreover, the targets of CDK-cyclin complex and the downstream transcriptional events are also evolutionarily conserved [1,4]. Among those, transcription factors and RNA polymerase II (Pol II) have been intensively studied. A few of the CDKs are involved in phosphorylation of Ser residues at the Pol II C-terminal domain (CTD), which is opposed by several CTD phosphatases. On the one hand, these evolutionarily conserved features have allowed scientists to harness the power of genetic and biochemical studies in model organisms including *S. cerevisiae* and the *Arabidopsis thaliana* model plant. On the other hand, yeasts, plants, and humans have been separated for more than a billion years of evolution. Therefore, humans and plants also have evolved unique mechanisms to enable them to adapt to various internal cues and external stimuli during growth and development.

**Figure 1:**
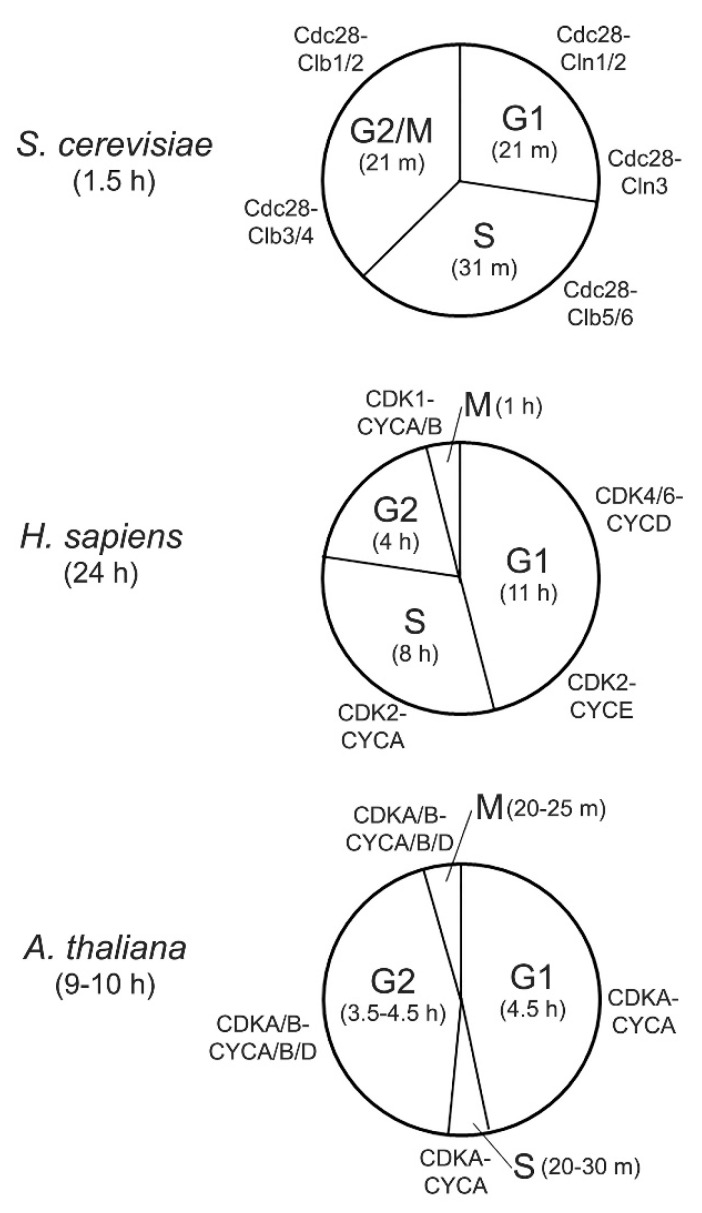
Varying lengths of cell cycle phases and the key CDK-cyclin pairs involved in each phase for budding yeast (mother cells), Arabidopsis plant (root cells), and human (cell culture). Of note, G2 and M phases in budding yeast largely overlap, and thus are conceptually merged. m stands for minute.

A comparison of cell cycle durations at each phase between *S. cerevisiae*, *A. thaliana*, and humans (*Homo sapiens*) reveals their similarities and differences (Figure 1). For budding yeast with a size of approximately 10 µm, a typical cell cycle is approximately 80–90 min. G2 and M phases largely overlap, and thus are conceptually merged. Although the G1 phase can be longer in relatively smaller, daughter cells (43 min) than in larger, mother cells (21 min), durations of S (26–31 min) and G2/M (28–30 min) phases do not have a big difference and are size-independent [5,6]. A typical human cell in culture, with a size of around 100 µm, has a cell cycle of approximately 24 h, with 11, 8, 4, and 1 h for G1, S, G2, and M phases, respectively [7]. Similar to human cells, the cell cycle lengths of plant cells, with a size range of 10–100 µm, can also vary, depending on the cell types. Recent studies of Arabidopsis root cells found that their cell cycles are in the range of approximately 9–10 h, which is similar in tomato, tobacco, and other plant species with bigger genomes than Arabidopsis [8]. While humans have a relatively longer G1 phase (46% of the total cell cycle duration) than budding yeast (23% of the cycle duration), the absolute time for DNA duplication in yeast is understandably shorter than in humans, given that the yeast genome (12 Mb) is considerably smaller than that in humans (3 Gb). However, relative to the total cell cycle duration, they both have a long S phase (i.e., approximately 1/3 of the total cycle duration). In contrast, plants have a very short S phase of approximately 20–30 min, which corresponds to approximately only 3–5% of the total cycle duration, although plants and humans have similar genome sizes, with 125 Mb in Arabidopsis and 4.5 Gb in tobacco. The reason for this difference in S phase lengths is not clear. Plants differ from humans and other animals in that plant cells have a cell wall that limits their mobility. Therefore, these sessile organisms have to develop unique mechanisms in order to respond and adapt to dynamic environmental conditions.

In this mini-review, I will first summarize the conserved features in cell cycle transcriptional control across the three eukaryotic kingdoms, Fungi, Plantae, and Animalia, Fungi, by highlighting some of the most important findings from studies of budding yeast, *Arabidopsis* plants, and human. These include CDKs, CTD phosphatases, and Pol II transcription regulated by these protein kinases and phosphatases. These CDKs and CTD phosphatases have conserved domains across the three kingdoms, but some of them also evolve with unique structure and function in each of the kingdoms. The unique aspects and potential challenges of transcriptional control in plant cell cycle will be discussed. For an in-depth review of recent updates in plant cell cycle control, readers are referred to several most recent, comprehensive reviews by other authors [9,10,11].

## 2. Cyclin-Dependent Kinases in Yeast, Human, and Arabidopsis

**Figure 2:**
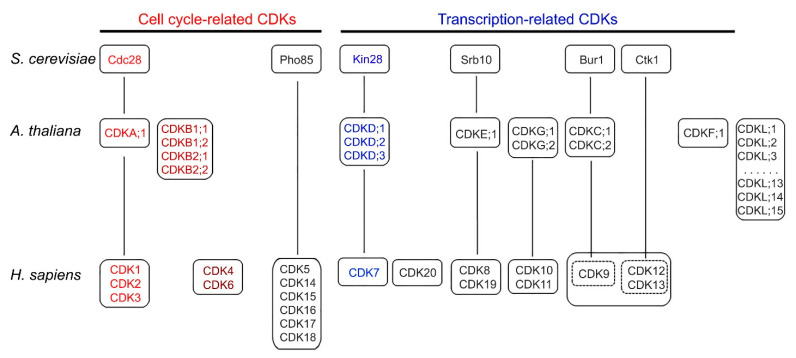
Groups of CDKs from yeast (*S. cerevisiae*), Arabidopsis (*A. thaliana*), and human (*H. sapiens*). Note that Arabidopsis contains 15 CDK-like genes (CDKL1 to CDKL15). The vertical line indicates an orthologous relationship. CDKs in color represent the two major regulators that are involved in cell cycle and transcriptional control.

CDKs represent a subgroup of the CMGC Ser/Thr kinases first discovered from the budding yeast model system for their important role in cell cycle control [3]. Their catalytic function requires an association with multiple cyclins, which act as a regulatory subunit. It seems that the same CDK associated with different cyclins act at distinct phases of cell cycle (Figure 1). Subsequent studies in budding yeast and other eukaryotes revealed that more CDKs are involved in gene transcription and they are bound by a single cyclin. Therefore, there are two types of CDKs, cell cycle-related and transcription-related CDKs. As presented in Figure 2, among a total of six CDKs in budding yeast, two (Cdc28 and Pho85) belong to cell cycle-related CDKs, while four other CDKs (Kin28, Srb10, Bur1, and Ctk1) are grouped into transcription-related CDKs [3].

Human and Arabidopsis plant contain a larger number of CDKs than budding yeast [3,9]. In humans, a total of 20 CDKs are divided into eight groups, with three groups (CDK1/2/3, CDK4/6, and CDK5/14/15/16) recognized as cell cycle-related CDKs and five other groups as transcription-related CDKs (Figure 2). CDK9 (the Bur1 ortholog) and CDK12/13 (orthologs of Ctk1) are regarded as the eighth group represented by CDK9. Furthermore, humans have three groups of CDKs (CDK4/6, CDK10/11, and CDK20) that are not orthologous to any yeast CDKs. Compared to slightly more than 6000 protein-coding genes in budding yeast and approximately 20,000 protein-coding genes in human, it seems that the number of human CDKs expanded in a similar scale as the whole set of protein-coding genes.

However, Arabidopsis genome has a significantly larger expansion of CDKs than human, with a total of 30 CDK and CDK-like (CDKL) genes [9,12,13], given only a slightly larger number of protein-coding genes in Arabidopsis (about 25,000) than human (approximately 20,000). These Arabidopsis CDK and CDKL genes can be separated into eight groups (Figure 2), and four of them are orthologous to both yeast and human, including CDKA;1, CDKD;1/2/3, CDKF;1, and CDKC1/2. It seems that the orthologs of yeast Cdc28 in human (CDK1/2/3) and Arabidopsis (CDKA;1) have played a similar and important role in controlling different phases of cell cycle (Figure 1). Note that the Arabidopsis CDKB group and the human CDK4 group are not orthologous to each other and to budding yeast, but they have been found to regulate the cell cycle, indicating the expansion of animal-specific and plant-specific CDKs in cell cycle control. While Arabidopsis does not have yeast Pho85 and Ctk1 orthologs, unlike human, CDKF;1 is unique to plants. In addition, Arabidopsis has 15 CDKL genes, which are not the orthologs of any of yeast and human CDKs.

## 3. Pol II CTD Phosphatases in Yeast, Human, and Arabidopsis

**Figure 3:**
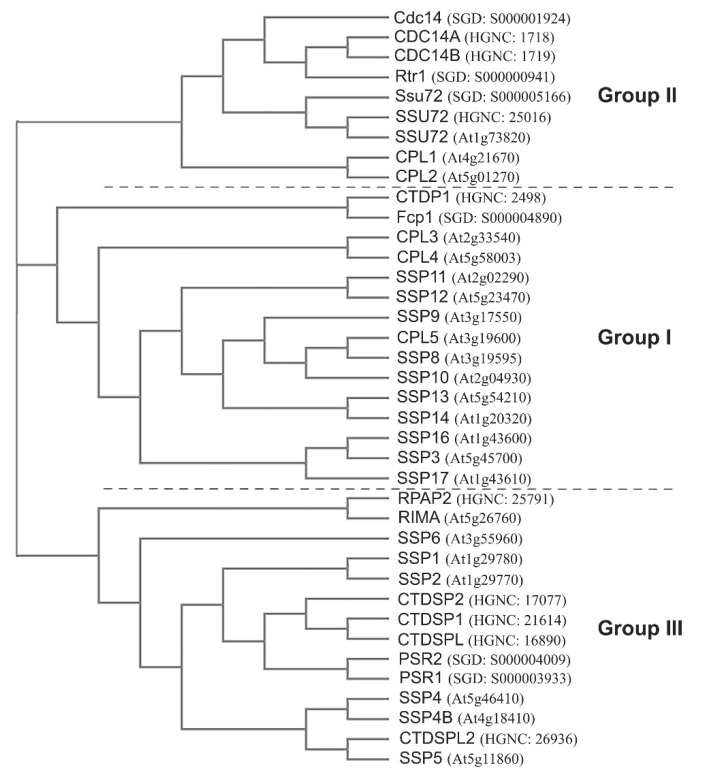
CTD phosphatase protein tree analysis. Full-length protein sequences of CTD phosphatases from yeast, Arabidopsis, and humans were used in the tree analysis. CTD phosphatases are separated into three groups (I, II and III). The gene ID for each protein was given in parenthesis for yeast (SGD), Arabidopsis (At), and humans (HGNC).

Protein phosphatases involved in dephosphorylation of Ser at 2, 5, and 7 positions in the highly conserved heptad peptide (Y_1_S_2_P_3_T_4_S_5_P_6_S_7_) repeat of the CTD of the largest subunit of Pol II have been identified in yeast, human, and Arabidopsis (Figure 3), and some of these phosphatases have been functionally characterized. The phosphorylated forms of Ser2, Ser5, and Ser7 are designated Ser2P, Ser5P, and Ser7P, respectively, in this review. Yeast contains four known CTD phosphatases (Fcp1, Rtr1, Ssu72, and Cdc14) and two probable phosphatases (PSR1 and PSR2) that share a sequence homology with another type of CTD phosphatases initially classified as small CTD phosphatases (SCP). Human has one ortholog for each of the three classical CTD phosphatases, FCP1 (renamed as CTDP1), RPAP2 (claimed to be orthologous to yeast Rtr1), and SSU72, but two orthologs for yeast Cdc14, CDC14A, and CDC14B. In addition, human contains four SCP members, renamed as CTD small phosphatase 1 (CTDSP1), CTDSP2, CTDSP-like (CTDSPL), and CTDSPL2.

Arabidopsis has the largest number of CTD phosphatases among these three eukaryotes. In the Arabidopsis genome, there are five CTD phosphatase-like (CPL) genes, CPL1 to CPL5 [14,15], and one ortholog for Ssu72 and RPAP2, respectively, designated SSU72 [16] and RIMA [17]. Surprisingly, Arabidopsis contains a large number of SCP1-like small phosphatases, designated SSP. Initially, a total of 18 SSP genes (SSP1–SSP18) were proposed [18]. However, the original SSP7 (At3g19600) was renamed as CPL5 [15], and a new member, SSP4B, was added [19]. An extensive genome sequence search revealed that the original SSP15 (At3g15330) is a pseudogene and that At3g19590 is also closely related to all other SSP proteins. In addition, the updated Arabidopsis genome does not have SSP8, which was originally proposed as a fused gene spanning the CPL5 (At3g19600) locus. In order not to cause any confusion in gene name designation for SSP [18], At3g19590 is proposed to encode SSP7, and SSP16 and SSP17 are kept without change. Therefore, there are a total of 16 SSP genes in Arabidopsis, *SSP1*–*SSP3*, *SSP4*, *SSP4B*, *SSP5*–*SSP7*, *SSP9*, *SSP11*–*SSP17* (Figure 3). Of note, only SSP4, SSP4B, and SSP5 have been shown to exhibit a CTD phosphatase activity [19], while 13 other SSP proteins are annotated as the haloacid dehalogenase-like hydrolase (HAD) superfamily proteins, with SSP7–SSP17 having a gene ontology (GO) term of dephosphorylation of Pol II CTD. Given that Fcp1, the founding member of FCP/SCP phosphatases, has a biochemical mechanism more closely resembling the HAD superfamily proteins [20], it is conceivable that the 13 other SSP proteins might also possess CTD phosphatase activity. Overall, relative to the total number of protein-coding genes in the yeast, Arabidopsis, and human genomes, CTD phosphatases in Arabidopsis have expanded considerably more than that in humans, a situation similar to CDKs. It remains to be determined whether this unequal expansion of CDK and CTD phosphatase genes in Arabidopsis is relevant to the unique aspects of plant growth and development.

The protein tree analysis of these CTD phosphatases and their closely related phosphatases indicates that they are classified into three groups (Figure 3). Group I contains Fcp1, its orthologs in human (CTDP1) and Arabidopsis (CPL3/4), and Arabidopsis CPL5 and other SSP proteins. Fcp1 and its orthologs possess an N-terminal FCP1 homology (FCPH) domain, a breast cancer protein-related C-terminal domain (BRCT), and a C-terminal region involved in the interaction with RAP74, a component of general transcription factor TFIIF [20]. The FCPH domain is important for phosphatase activity, while the BRCT domain is involved in protein-protein interaction. Group II contains CPL1/2, SSU72, Cdc14, and Rtr1. This group of CTD phosphatases acts to dephosphorylate Ser5P (although some of them also impact Ser2P dephosphorylation when mutated) and are structurally more diverse, with Rtr1 considered as an atypical CTD phosphatase (due to its weak in vitro CTD phosphatase activity and the lack of apparent CTD phosphatase domain). Indeed, the functional ortholog of yeast Rtr1 in human, RPAP2, is placed in Group III. SSU72 and Cdc14 in yeast and human are Ser5 phosphatases, but they can also dephosphorylate Ser2P (Cdc14) or Ser7P (SSU72). However, the CTD phosphatase activity of Arabidopsis SSU72 has not been reported, and there is no Arabidopsis ortholog for Cdc14. Arabidopsis CPL1 and CPL2 are unique in that in addition to a CTD phosphatase domain, they also contain one (for CPL2) or two (for CPL1) double-stranded RNA binding motifs [21]. CPL1/2 possess Ser5P-specific phosphatase activity in vitro [22], but our in vivo studies found that the Ser2P level is elevated in both single gene mutants and the double mutants [23]. Group III includes human RPAP2 and its Arabidopsis ortholog RIMA, the biochemically validated small CTD phosphatases in human (CTDSP1/2/L) and Arabidopsis (SSP4/4B/5), and all other putative SSP proteins in Arabidopsis (SSP1/2/5/6) and yeast (PSR1/1). RPAP2 and CTDSP1/2/L in human and SSP5 in Arabidopsis have been demonstrated to dephosphorylate Ser5P specifically. In addition, Arabidopsis SSP4 and SSP4B can dephosphorylate Ser2P [19]. Overall, the phosphatases for the critical Ser residues in the Pol II CTD have been identified, with diverse structural and functional conservation, as well as divergence.

## 4. Control of Transcription during the Cell Cycle

### 4.1. Importance of Precise, Global Transcriptional Control in Cell Cycle

Progression through the cell cycle requires synthesis of more than 1000 cell cycle-dependent or cell cycle-related proteins [1,24]. This process is regulated at the transcriptional level primarily during the S and M phases. During the S phase, a group of genes encoding proteins important for DNA replication and DNA repair are transcribed, while another group of genes that encode proteins involved in mitosis and cytokinesis are expressed during the M phase [24]. Then, these cell cycle phase-specific proteins are subjected to proteasome-mediated degradation after completion of the S phase and the M phases, respectively, to ensure precisely ordered progression through the cell cycle and eventual exit from the cycle until mitotic cues are perceived to initiate another round of cell division.

Transcription of all protein-coding and many non-coding RNA genes requires the function of RNA polymerase II (Pol II) together with key transcription factors. While Pol II binds to the core promoter (which is relatively conserved among numerous genes) and thus functions in basal-level, global transcription, sequence-specific transcription factors control the transcription of genes with the corresponding enhancer sequence in the right tissues or cells and at the right time. Intensive studies of transcriptional control during G1-S and G2-M transitions have led to a consensus that a combination of several groups of transcription factors or complexes dictate the phase-dependent expression of genes [1]. These transcription factors include the RB pocket protein family, the E2F small transcription factor family, and MuvB complexes [1]. The details of the dynamic control are described in a comprehensive review [1]. In brief, upon activation, E2F transcription factors recognize the E2F binding elements present in G1-S transition- or S phase-related genes to turn on the expression of these target genes. For transcription of the G2-M transition or M phase-specific genes, the activating B-MYB and FOXM1 transcription factors, respectively, form the complexes with MuvB, which are then recruited to the CHR promoter elements of G2/M phase-related genes. In other phases, when those genes do not need to be transcribed, distinct repressor complexes (such as DREAM) are recruited to the E2F or CHR promoter elements. Through this mechanism, the precise timing of cell cycle-dependent transcription can be achieved to ensure the orderly progression of various phases in the cell cycle.

### 4.2. Pol II CTD Phosphorylation Is Controlled by CDKs and CTD Phosphatases

Pol II is a large multi-unit protein complex, with its largest subunit RPB1 as the core of Pol II transcriptional machinery. The CTD of RPB1 contains various numbers of highly conserved heptad peptide (Y_1_S_2_P_3_T_4_S_5_P_6_S_7_) repeat, ranging from 26 in budding yeast and 29 in fission yeast to 34 in Arabidopsis and 52 in human [20,25,26,27,28,29,30]. Each of the seven amino acids in the repeat may undergo different modifications (e.g., S/T/Y phosphorylation, S glycosylation, and P isomerization), and each repeat may have a different posttranslational modification pattern [31]. Therefore, the CTD potentially exhibits a large and complex pattern collectively called the CTD code [20,25,26,28,29,30,31]. The CTD code, in particular the levels of Ser2P, Ser5P, and Ser7P during three stages of transcription, is critical for transcriptional control. In general, before transcription starts, Ser2, Ser5, and Ser7 are all unphosphorylated, and the initiation of transcription requires Ser5P. However, the Ser5P level declines when gene transcription enters the elongation stage. In the meantime, Ser2P and Ser7P levels increase during productive elongation, and they all decrease at the termination stage, in order that the Pol II CTD enters another transcriptional cycle.

The dynamic CTD phosphorylation pattern during transcription is tightly regulated by various CDKs and CTD phosphatases [20,25,26,27,28,29,30]. In vitro and in vivo biochemical studies, together with genetic evidence, have shown that most of CDKs and CTD phosphatases have targeted two or three Ser positions [20,26,27,32]. The result is summarized in Table 1. Note that overall, orthologs of CDKs and CTD phosphatases in yeast and human have almost identical Ser-specificity. However, Arabidopsis orthologs of CDKs and CTD phosphatases have slightly different Ser specificity, except for CDKA1, SSU72, and RIMA, whose CTD Ser phosphorylation or dephosphorylation activity has not been reported yet. For example, CDKD;1/2/3 also phosphorylate Ser2, while yeast and human counterparts do not. In contrast, while Arabidopsis CDKC;1/2 are specific to Ser2P, their yeast and human orthologs phosphorylate Ser at all three positions (although they predominantly phosphorylate Ser2).

### 4.3. Transcriptional Control: Global vs. Centralized?

Given the large-scale gene expression needed to fulfil the distinct tasks of the S and M phases, two conflicting models have been proposed to explain the precise timing of phase-specific transcriptional control: The centralized, autonomous CDK-APC/C oscillator vs. the global transcriptional oscillator [33]. In the CDK-APC/C model, CDKs act to oppose the anaphase-promoting complex/cyclosome (APC/C, which possesses E3 ubiquitin ligase activity to degrade cyclin) and trigger phase-specific events, including phosphorylation of transcription factors. In turn, this precisely times the transcription of many phase-specific genes. The global transcriptional oscillator model [34] was proposed based on the findings on the yeast transcription factor network. In this model, transcription factors transcribed during one cell-cycle phase can bind the promoters of the next set of transcription factors that control phase-specific transcription.

The strongest evidence supporting the centralized CDK-APC/C oscillator model came from a single-cell study that observed time-series transcriptome changes during different phases of the budding yeast cell cycle in the B-cyclins (*CLN2*, *CLB2*, *Swi5*) mutants with “on” and “off” switches for controlling these individual cyclins [35]. The resulting transcription data were inconsistent with the global transcriptional oscillator model, and thus it was proposed that the CDK-APC/C oscillator predominantly entrains periodic cell cycle transcription. However, not all phase-specific genes were studied, and some of those genes under study did not exhibit a consistent transcriptional pattern.

In an effort to address which of the two models more likely operates in periodic control of transcription during the cell cycle, Cho et al. [33] analyzed transcriptome data using the yeast mutants depleted of B-cyclins and the *cdc14* and *cdc15* mutants as well. They found that a large subset of the cell cycle transcriptional program continued to oscillate in those yeast mutants arrested with constitutive Clb-CDK activity, which is inconsistent with the APC/C oscillator model. However, CDKs are required to maintain amplitudes of global transcriptional oscillations [35]. To reconcile these findings, Cho et al. [33] proposed an integrated CDK-APC/C and transcription factor network model. In this refined model, a global transcription oscillator drives periodic transcription, but CDKs are highly interconnected with transcription factors and contribute to robust, high-amplitude oscillations.

While this integrated model explaining how the CDK-APC/C oscillator and transcription factor network work together is attractive, it remains unknown whether Pol II itself is actively involved or simply serves as a machinery for basal transcription during precisely timed progression in the cell cycle. A recent study used the single-cell and single-molecule mRNA fluorescence in situ hybridization (smFISH) approach to count the number of mRNA molecules per cell in each phase of budding yeast cell cycle [36]. Their result surprisingly showed that all of the three main G1-S transition genes tested (*SIC1*, *CLN2*, and *CLB5*) had basal expression throughout the cell cycle. In contrast to the findings obtained using cell population, this single cell-based result indicates that these genes are not simply turned on or off completely, but instead they are expressed at high or low levels. While the biological relevance of this contrasting expression pattern needs to be further investigated, it is important to distinguish the possibilities whether this ubiquitous basal expression is under the control of the integrated CDK-APC/C and transcription factor network model or simply a reflection of Pol II basal activity.

## 5. Functional Conservation of CDK and CTD Phosphatases

In this section, three aspects of functional conservation for CDK and CTD phosphatases are discussed: Conserved CDKs in cell cycle and transcription, common substrates RB and E2F, and pathways leading to Pol II CTD Ser phosphorylation. Other aspects of functional conservation, such as the involvement of CDK activators (CAK) and inhibitors (CKI, Kip1, and Sic1) and the APC/C in regulation of CDK activities, can be found in other excellent comprehensive reviews [1,3,9].

### 5.1. CDK-Cyclin in Cell Cycle and Transcription

Ser/Thr kinase activity of CDKs is dependent on their regulatory subunit cyclin. There are more cyclin genes than CDKs, with 22 in budding yeast, 29 in human, and a significantly expanded number (at least 50) in Arabidopsis [3,9,37]. Therefore, one would expect that the large number of CDK-cyclin combinations would enable complex multicellular organisms, such as Arabidopsis and human, to undergo a wide range of growth and developmental behaviors in response to dynamic cues. However, the basic function of CDK-cyclin in cell cycle control is conserved although with certain degree of diversity in the regulatory patterns, which are contributed from other CDKs and their cyclin subunits [4]. As depicted in Figure 1, a single CDK (Cdc28) in yeast is sufficient to drive the progression of each phase during the cell cycle, with distinct cyclins at each phase. Cdc28 contains a conserved PSTAIRE motif in the cyclin binding domain. In the human cell cycle, almost all Cdc28 orthologs (CDK1/2/3) that also contain the PSTAIRE motif are involved, with CDK1 alone sufficient to drive the entire cell cycle and distinct CDK-cyclin pairs that predominantly function at different phases, such as CDK2-CYCE at late G1, CDK2/CYCA at S, and CDK1-CYCA/B at late G2 and M phases. In addition, other types of cell cycle-related CDKs, CDK4/6-CYCD, are also important at the early G1. However, only CDK1 seems to be essential, since the knockout of the CDK1 ortholog in mouse caused lethality, while *cdk2/4/6* triple knockout mice were still viable [38,39]. In Arabidopsis, CDKA1;1, the ortholog of yeast Cdc28 and human CDK1, also contains the PSTAIRE motif and has kinase activity peaked at the G1-S and G2-M transitions [9], suggesting its critical role in controlling the entire cell cycle (Figure 1). However, the *cdka;1* null mutant was still viable although the mutant was severely impacted, and the *cdka;1 cdkb1;1 cdkb1;2* triple mutant caused the cell cycle arrest [40]. Indeed, CDKBs and their cyclins are involved together with CDKA;1 in the control of the cell cycle (Figure 1). CDKBs have two subgroups, each with two members (Figure 2), and they all contain altered PSTAIRE motifs (PPTALRE in CDKB1 and P[S/P]TTLRE in CDKB2 subgroups). Due to this structural difference and the observation that Arabidopsis CDKBs could not complement yeast *cdc28* or *cdc2* mutants [40], CDKBs are considered a plant-unique group. Taken together, it seems that although human and Arabidopsis have evolved with an expansion of several cell cycle-related CDKs compared to yeast, the canonical PSTAIRE motif-containing CDKs (Cdc28, CDK1, and CDKA;1) have a conserved function in controlling the entire cell cycle. Nevertheless, the role of cell cycle CDKs, Cdc28, and CDK1, has been expanded to transcriptional control in yeast and human. Human CDK1 (previously called CDC2) can phosphorylate Pol II CTD Ser2 and Ser5 in vitro [41], and yeast Cdc28 only phosphorylates Ser5 [42]. This activity is believed to stimulate the Pol II basal transcriptional machinery to boost transcription of a subset of housekeeping genes upon entrance into the cell cycle [43]. However, it remains unknown whether this dual role in cell cycle and transcription is also conserved in Arabidopsis CDKA;1.

Another functionally well conserved CDK is CDK7, which is mainly involved in Pol II CTD phosphorylation and thus transcriptional control during the cell cycle. As discussed above, human CDK7 and its yeast ortholog Kin28 are believed to phosphorylate Ser5 and Ser7, but not Ser2 based on in vitro biochemical studies [44], while Arabidopsis counterparts CDKD1;1/1;2/1;3 also phosphorylate Ser2P, in addition to Ser5 and Ser7 [45]. However, using a potent and specific CDK7 inhibitor THZ1, we found a dramatic decrease of Ser2P, Ser5P, and Ser7P in human cells [46]. Despite this functional divergence, human CDK7 has emerged as a critical target in containing uncontrolled cell division in various tumor types [47,48]. CDK7 is a member of the general transcription factor TFIIH complex composed of 10 subunits. Phosphorylation by CDK7 of Ser5 at the hypophosphorylated Pol II CTD leads to transcriptional initiation and clearance from the promoter [48]. In addition, CDK7 also phosphorylates CDK9, which then becomes active to phosphorylate Ser2 of Pol II CTD, enabling productive transcription. Therefore, CDK7 has been considered as a key transcriptional CDK in Pol II control of transcriptional cycle, although CDK7 is completely dispensable for global transcription [48,49]. Consistent with the critical importance of CDK7 in human cell cycle transcription, inactivation of mouse *CDK7* [49] and yeast *Kin28* [50] led to cell cycle arrest, and the Arabidopsis triple mutant *cdkd;1 cdkd;2 cdkd;3* exhibited severely impacted plant growth [45]. Furthermore, studies in yeast have shown that Cdc28 cooperates with Kin28 to achieve full Ser5P in the Pol II CTD. Therefore, Kin28-mediated CTD Ser5P serves as a priming site for recruitment of Cdc28 to Pol II, linking the two most important CDKs, Cdc28/CDK1 and Kin28/CDK7, which are commonly perceived as cell cycle-related and transcription-related CDKs, respectively, to achieve the precise control of productive transcription during progression through the whole cell cycle [42,43].

### 5.2. Substrates RB and E2F

Accumulating evidence suggests that CDK control of the G1-S transition is more conserved in yeast, human, and Arabidopsis than in the G2-M transition. The G1-S phase transition in human cell cycle is mainly controlled by CDK2-CYCE and CDK2-CYCA (Figure 2) that regulate two opposing transcriptional regulators, RB and E2F. RB was identified as a tumor suppressor gene from a retina cancer called retinoblastoma, while E2F is a small transcription factor family. When RB binds to E2F1–3, the RB-E2F complex is formed via the dimerization partner DP, which inhibits the E2F1–3 activity, and thus transcription is repressed. At the late G1 phase, CDK2 phosphorylates RB, which becomes inactive but releases E2F1–3 [1,51]. Ultimately, genes required for DNA synthesis and DNA repair are transcribed. At the late M phase, RB is dephosphorylated, and thus binds to and inhibits E2F, which then inhibits transcription until a new round of cell division is executed. Homologs of RB and E2F have been identified in yeast and Arabidopsis: Whi5 (functional homolog of RB with no sequence homology) and SBF, respectively, in yeast [51], and RBR and E2F in Arabidopsis [52,53]. Arabidopsis RBR contains several domains similar to RB, and it has been shown to be the CDKA;1 target in the G1-S transition [40]. In Arabidopsis, there are at least six E2F genes (*E2F1–3* and *E2FA-C*) and several E2F-like or atypical E2F genes [52]. Despite some divergence between Arabidopsis and yeast or human, it seems that the double-negative regulatory feedback loops between CDK and RB/Whi5/RBR are conserved in these three eukaryotes [4]. Therefore, the findings that the substrates (Whi5/RB/RBR) of cdc28/CDK1/CDKA;1 and the associated transcription factors (SBF/E2F) are also functionally conserved suggest that these three canonical CDKs containing the PSTAIRE motif act as universal regulators of the cell cycle with a conserved biochemical mechanism.

### 5.3. Substrate Pol II CTD and Its Upstream Regulatory Pathways: Classical vs. Shortcut?

**Figure 4:**
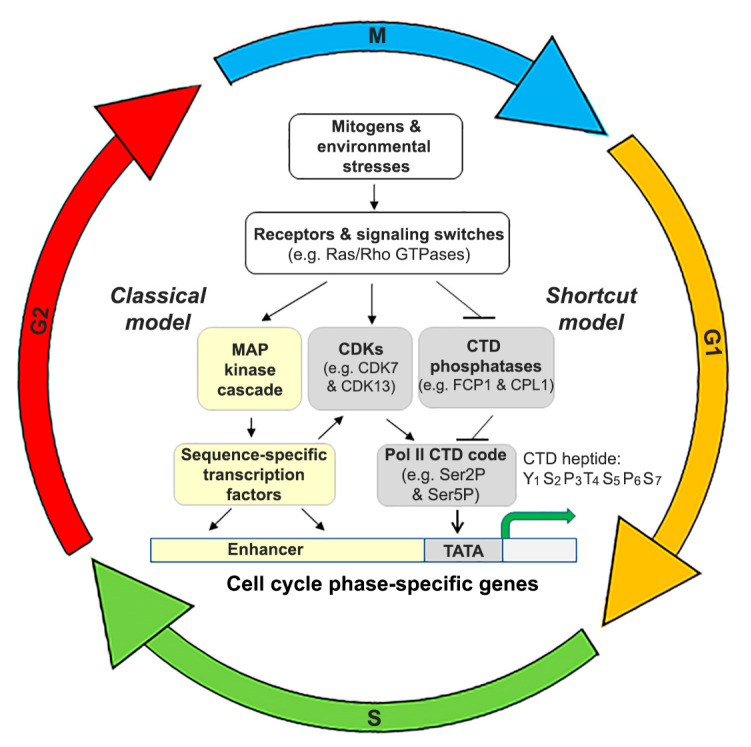
A proposed cooperative mechanism of Pol II transcriptional control via integration of the classical and shortcut models. Spatial and temporal transcription control of cell cycle phase-specific genes is conferred by sequence-specific transcription factors, which are activated by the classical model and consequently bind to the enhancer of those cell cycle genes. Direct modulation of the Pol II CTD code via regulating the abundance or activity of CDKs and CTD phosphatases allows Pol II, which recognizes the core promoter including the TATA box, to undergo productive transcription. Together, this cooperative mechanism by integrating the two intracellular signaling models enables a cell to quickly achieve the most precise and productive control of large-scale transcription critical for completing each phase of the cell cycle.

The highly conserved heptad peptide repeats in the CTD of Pol II are dynamically regulated by several CDKs and CTD phosphatases in response to mitogenic signals. Overall, CDKs and CTD phosphatases in yeast, human, and Arabidopsis have similar CTD Ser specificity, although some of the orthologs have more or less Ser specificity (Table 1). How these kinases and phosphatases are controlled by upstream signals in gene expression regulation have received increasing attention. Accumulating evidence suggests the existence of two models of transcriptional control (Figure 4) [54]. In the “*classical*” model of transcriptional control, which is frequently described in molecular genetics or cell biology textbooks, extracellular proliferation cues first activate intracellular signaling switches, such as the well-studied Ras and Rho families of small GTPases, which in turn activate the MAP kinase cascade. Subsequently, phosphorylated MAPKs phosphorylate various sequence-specific transcription factors, which then become active and bind to the gene-specific enhancer and consequently, recruit Pol II, by interacting with the general transcription factor TFIIH complex, to the core promoter of those genes to be transcribed [54]. Moreover, this interaction stimulates Ser5P in the CTD of RPB1 via activation of CDK7 present in the general transcription factor TFIIH complex and/or the mediator complex [55,56,57]. Ultimately, transcription is initiated. Since many components and steps are indirectly involved in the Pol II CTD Ser5P, this classical model is also called the indirect model and has been considered an intracellular signaling paradigm.

The other model, called a *shortcut* model, depicts the direct modulation of Pol II CTD Ser5P and Ser2P status by Ras GTPase-exerted PKA signaling to the mediator component Srb9 in yeast [58] or by Rho GTPase-mediated degradation of CTD phosphatases in yeast and Arabidopsis [23]. Importantly, we have found that the Rho signaling shortcut to Pol II CTD Ser2P and Ser5P was controlled by proteasome-mediated degradation of CPL1 and CPL2 in Arabidopsis or Fcp1 in yeast [23]. Furthermore, Rho family GTPases (Cdc42 and Rac1) in human cells also seem to suppress CTD phosphatases in a GTPase-specific manner: Suppression of RPAP2 by Cdc42 signaling but not Rac1, and suppression of CTDP1 (FCP1) by Rac1 signaling but not Cdc42 [46]. This strongly suggests that the shortcut model of Pol II transcription is conserved from yeast to Arabidopsis and human. In addition to the control of CTD phosphatases, CDKs (for example, CDK7 and CDK13) are also activated by Rho signaling, although these two CDKs do not exhibit any specificity for Rac1 and Cdc42, as the knockdown of both GTPases by RNA interference reduced the levels of these two CDKs.

What is the implication for the existence of both classical and shortcut models in Ras or Rho GTPase signaling to Pol II transcription across three eukaryotic kingdoms? Here, a cooperative control hypothesis is proposed (Figure 4). Since Pol II CTD can be directly targeted by signaling pathways in the shortcut model, rather than via the MAP kinase cascade in the classical model, the shortcut model has the advantage of rapidly bringing up large-scale gene expression changes in response to urgent growth or proliferation cues. Yet, the spatial and temporal control of transcription for those cell cycle-related genes depends on those sequence-specific transcription factors. Therefore, in response to a signal for cell division, a cell can activate Rho or probably other signaling molecules as well. In addition, a cell can use the classical model to promote the precise binding of sequence-specific transcription factor to the enhancer sequence of the cell cycle-related genes required at each phase, and in the meantime, it also can use the shortcut model to quickly modulate the Pol II CTD phosphorylation code. Therefore, this cooperative mechanism enables a cell to quickly achieve the most precise and productive control of large-scale transcription. This mechanism may be essential for cellular organisms to efficiently complete a cell cycle and determine whether additional rounds of cell division are needed when facing dynamic internal cues and external stimuli.

## 6. Functions of CDKs and CTD Phosphatases Unique to Arabidopsis

As discussed above, Arabidopsis plants have expanded the families of CDKs and CTD Ser phosphatases dramatically compared to yeast and human. Moreover, although the Arabidopsis orthologs of CDKs and CTD phosphatases have conserved functions as in yeast and human cell cycle control, they also exhibit some diversification in performing their biochemical functions or participating in cell cycle progression. Therefore, in order to understand why plants evolved with many CDKs and CTD phosphatases, it is important to address the key question: What are the functions for those plant-specific CDKs and CTD phosphatases in relation to cell cycle control?

### 6.1. Plant-Specific CDKs

Since the identification of two CDK genes (*cdc2a* and *cdc2b* now called *CDKA;1* and *CDKB1;1*, respectively) from Arabidopsis 30 years ago [59,60], functions of many CDKs and their cyclin partners have been reported. Overall, we have more knowledge of CDKs than CTD phosphatases regarding cell cycle control. For the functional details of individual CDKs, including those plant-unique CDKs, readers are referred to prior reviews [27,61,62] regarding the cell cycle or transcriptional control or the three most recent, comprehensive reviews [9,10,11] regarding other biological processes, such as growth and development, hormone response, and nutrient or biotic/abiotic stress response. Here, only the plant-specific CDKF, CDKG, and CDKL genes in Arabidopsis are summarized in relation to their cell cycle transcriptional control.

CDKF;1 is a plant-unique CDK activating kinase (previously called CAK1) that can phosphorylate two other CAKs now designated as CDKD;2 and CDKD;3, but not CDKD;1 [63]. Although when it was first identified and characterized, CDKF;1 was believed not to phosphorylate Pol II CTD based on the fractionation assay [64], a subsequent in vitro study demonstrated that it specifically phosphorylates Ser7 [45]. However, the *cdkf;1* knockout mutant also had lower levels of Ser2P and Ser5P than the wildtype during later stages of seedling development, but not in 7-day-old young seedlings [45]. The *CDKF;1* transcript level gradually increased during seedling development, suggesting that CDKF;1 is developmentally regulated. However, the alteration of Ser2P could not be explained simply by the loss of function in CDKD group kinases, which phosphorylate all three Ser resides in the Pol II CTD, given that the Ser2P level was even lower than in the *cdkd;1 cdkd;2 cdkd;3* triple mutant [45]. Moreover, genetic evidence suggests that CDKF;1 and CDKDs have slightly different functions, in which CDKF;1 plays a distinct role, mainly in post-embryonic development, while CDKD;1 and CDKD;3 function as CAKs in the control of mitosis [65]. Therefore, it was proposed that CDKF;1 is also required for regulating CDKD-independent Ser2 kinase activity [27,45]. In addition to phosphorylating CDKDs, CDKF;1 was also found to phosphorylate and activate CDKA;1 in Arabidopsis root protoplasts [66]. However, genetic evidence shows that CDKF;1 is dispensable for CDKA;1 activation [67]. Therefore, CDKF;1 is suggested to play a more important role in CDK phosphorylation than in CTD phosphorylation. Consistent with its role as CAK for both cell cycle-related CDKA;1 and transcription-related CDKDs and as a CTD kinase, knockout of *CDKF;1* led to the formation of curling serrated leaves, arrested root growth, and severe dwarfism, which were caused by the decreased cell number and cell size [45,67]. Therefore, genetic and biochemical evidence support the fact that CDKF;1 is a major regulator of cell proliferation, although it remains unknown whether CDKF;1 acts at a specific cell cycle phase or throughout the cell cycle. The fact that *CDKF;1* expression did not seem to considerably change during the cell cycle progression indicates that it is likely regulated at the translational or kinase activity level during the cell cycle progression [13].

CDKG;2 has been found to regulate RNA splicing [68,69,70], similar to its counterparts in human (CDK10/11). However, CDKG;2 acts in different biological processes than CDKG;1. *CDKG;1* is essential for synapsis and recombination during male meiosis [71,72], while *CDKG;2* is involved in flowering and response to heat and salt stresses [68,69,73]. Interestingly, the *CDKG;1* transcript level decreased from G1/G0 to S and G2, although *CDKG;2* expression stayed almost the same during cell cycle progression [13]. Therefore, it will be important to determine whether the CDKG;1-controlled meiosis is related to the transcriptional control of cell cycle-related genes.

The role of 15 CDKL genes has not been extensively studied. Using synchronized Arabidopsis cell cultures to survey expression profiles of Arabidopsis core cell cycle regulators [13], it was found that several *CKL* (now called *CDKL*) genes exhibited cell cycle phase-correlated expression patterns. For example, when re-entering the cell cycle, *CDKL;3* had a gradual increase from G0/G1 to S, but then decreased at G2, while *CDKL;5* and *CDKL;6* expression decreased overall from G0/G1 to S and G2. *CDKL;3* was also identified from a mutant impaired in beta-aminobutyric acid (BABA)-induced sterility (*ibs1*), but how its kinase activity is involved in cell cycle progression and whether this role is related to priming for defense gene expression remain to be determined [74,75]. In addition, two *CDKL* genes are specifically (*CDKL;1*) [76] or preferentially (*CDKL;15*) [13] expressed in flowers, but their expression did not change dramatically during cell cycle progression [13].

### 6.2. Plant-Specific CTD Phosphatases

Among the three groups of CTD phosphatases, several members of Group I (CPL3, CPL4, CPL5) and Group III (SSP4, SSP4B, SSP5, and RIMA) have been functionally characterized [14,15,17,19,77,78,79,80,81]. These include Pol II CTD Ser dephosphorylation activity (except for CPL3 and RIMA), which is similar to their orthologs or closest homologs in yeast and human. When these genes are inactivated or overexpressed, they exhibited phenotypic alterations in hormone, nutrient, biotic, and abiotic stress responses. However, none of the mutants or transgenic lines are characterized regarding their cell division phenotype. Group II contains an SSU72 ortholog, which is shown to act in flowering time control [16], although whether it possesses CTD phosphatase activity remains unknown. Therefore, CPL1 and CPL2, which belong to a unique subgroup within Group II (Figure 3) due to the presence of RNA binding motifs not found in any other CTD phosphatases, represent unique plant-specific CTD phosphatases with a likely involvement in cell division [23], as discussed in detail below.

*CPL1* was first identified from a genetic screen as allelic mutants showing high expression of a presumably stress responsive *RD29A-Luciferase (LUC)* reporter gene [21,82], and *CPL2* was then found based on the sequence homology with CPL1. CPL1 was found to dephosphorylate Ser5 specifically in vitro [22], but the loss-of-function and gain-of-function of *CPL1* alleles led to a consistent increase or decrease of both Ser5P and Ser2P [23]. Therefore, it remains to be clarified whether the observed Ser2P impact is due to an indirect effect caused by genetic perturbation of *CPL1* or whether the lack of Ser2 dephosphorylation by CPL1 is due to the lack of a critical cellular factor in the in vitro biochemical assay. Surprisingly, *CPL1* was frequently identified from various mutant screens, including from mutants showing increased expression of reporter genes, such as silenced *miRNA-LUC* [83], salt-inducible *SOT12-LUC* [84], cold-inducible *CBF2:LUC* [85], and disease responsive *GSTF8:LUC* [86], or from an enhancer of CA-rop2 (constitutively active form of ROP2) in cell shape [23]. Together with other phenotypes in the *cpl1* mutants, such as iron deficiency response [87] and floral transition [88], these lines of genetic evidence demonstrate CPL1 as a critical and global regulator in growth, development, and stress responses. Consistent with its role in transcriptional control, expression of many genes is affected in the *cpl1* mutants [23,83,86,87]. Of note, CPL1 is frequently regarded as a negative regulator of transcription due to its dephosphorylation impact on Pol II CTD Ser2 and Ser5. However, the aforementioned transcriptomic studies revealed that a similar set of genes are upregulated and downregulated. Therefore, the differing roles of the plant-unique CPL1 and probably CPL2 in transcriptional control may be context-dependent or due to multiple regulators that are influenced by CPL1 and CPL2.

As only our recent study observed the cell number increase and the cell size decrease during cotyledon development in the *cpl1 CA-rop2* background or the *cpl1 cpl2* double mutants [23], there is no considerable knowledge regarding how CPL1/2 control the cell cycle. Based on the biochemical and genetic evidence, we only know to date that they are inhibited by the signaling of Arabidopsis ROP family GTPases via a proteasome-mediated degradation mechanism. Moreover, a major growth hormone auxin, which has been shown to activate ROP GTPases [89,90], also stimulates Pol II CTD Ser2P and Ser5P in a ROP2/4-dependent manner [23]. Therefore, auxin-exerted gene expression probably involves the shortcut model described above to achieve the rapid and large-scale transcriptome changes needed for cell growth. As ROP GTPases act as a universal signaling switch for multiple hormones or developmental processes and various stress responses, it remains to be determined how the ROP2/4-CPL1/2 signaling shortcut is involved in many different processes. The finding that yeast Cdc42 GTPase signaling also promotes Fcp1 degradation [23] and the observation that human Rac1 and Cdc42 activity differentially inhibits CTDP1 (FCP1) and RPAP2 [46] may provide clues to this signaling specificity. For example, the shortcut model may involve different members of Rho GTPases and target any of three groups of CTD phosphatases (and CDKs, as well). Together with sequence-specific transcription factors, these complex interplays will enable plants to precisely time the expression of genes required for cell division and other biological processes.

## 7. Future Perspectives

Studies at the genetic, biochemical, and system levels from the simple unicellular yeast model to complex eukaryotes such as Arabidopsis model plants and humans have started to reveal the mechanisms for the precise control of phase-related transcription during the cell cycle progression. One question that remains to be answered is: Why do Arabidopsis plants with a considerably smaller genome size than humans evolve with substantially more CDKs and CTD phosphatases? A few possibilities have been proposed to explain the uniqueness of plant structure and function, including a bigger demand for these sessile organisms to respond and adapt to dynamic environmental challenges, and consequently with a high degree of developmental plasticity. However, molecular details are needed to answer this question and ultimately will contribute to our mechanistic understanding of convergence and divergency in the transcriptional control that makes an organism decide to enter the cell cycle or not and if that is the case, complete the entire cell cycle without delay and error. Therefore, many prior functional studies of genes, which have been emphasized at the organismal level, need to be assessed with regards to cell cycle progression. Moreover, given the consensus that individual cells frequently deviate from the population of cells, the use or development of single-cell technologies that minimally disturb the physiological state of cells, coupled with single-molecule techniques to count the individual cell cycle-related mRNA per cell, will be critical. Another challenge lies in the functional redundancy of many cell cycle-related genes in Arabidopsis and human (and the rodent or primate animal models, as well), such as several CDKs, cyclins or CTD phosphatases even within a subgroup. While some specific CDKs have been shown to regulate distinct cell cycle phases, it will be interesting to determine whether CTD phosphatases act in the same fashion during the cell cycle progression. Thus, functional redundancy and specification of these cell cycle transcriptional regulators need to be dissected using CRISPR/Cas9-based multiplex genetic manipulation. With these advanced single-cell and genome editing tools, the cooperative transcriptional mechanism, which is proposed here and involves sequence-specific transcription factors as well as the shortcut model of Pol II CTD code modulation (via CDKs and CTD phosphatases), can be tested and refined. Ultimately, a complete regulatory network can be assembled, which governs how individual organisms quickly achieve the most precise and productive, large-scale transcription of phase-specific genes required for orderly progression throughout the entire cell cycle.

## Figures and Tables

**Table 1:** CDKs and CTD phosphatases with known CTD Ser activities in yeast, Arabidopsis, and human. * Denotes the CTD phosphatases not yet studied regarding their Ser dephosphorylation activity.

	*S. cerevisiae*	*H. sapiens*	*A. thaliana*	Ser-Specificity
**CDKs**	Kin28	CDK7		Ser5, Ser7
			CDKD;1, CDKD;2, CDKD;3	Ser2, Ser5, Ser7
	Bur1	CDK9		Ser2, Ser5, Ser7
			CDKC;1, CDKC;2	Ser2
	Ctk1	CDK12, CDK13		Ser2, Ser5, Ser7
			CDKF;1	Ser7
	Cdc28		CDKA;1 *	Ser5
		CDK1		Ser2, Ser5
**CTD Phosphatases**	Fcp1	CTDP1	CPL3 *, CPL4	Ser2, Ser5
	Ssu72	SSU72	SSU72 *	Ser5, Ser7
	Rtr1	RPAP2	RIMA *	Ser5
	Cdc14	CDC14A, CDC14B		Ser2, Ser5
		CTDSP1, CTDSP2, CTDSPL	SSP5	Ser5
			SSP4, SSP4B	Ser2, Ser5
			CPL5	Ser2
			CPL1, CPL2	Ser2, Ser5
